# Data analysis guidelines for single-cell RNA-seq in biomedical studies and clinical applications

**DOI:** 10.1186/s40779-022-00434-8

**Published:** 2022-12-02

**Authors:** Min Su, Tao Pan, Qiu-Zhen Chen, Wei-Wei Zhou, Yi Gong, Gang Xu, Huan-Yu Yan, Si Li, Qiao-Zhen Shi, Ya Zhang, Xiao He, Chun-Jie Jiang, Shi-Cai Fan, Xia Li, Murray J. Cairns, Xi Wang, Yong-Sheng Li

**Affiliations:** 1 State Key Laboratory of Reproductive Medicine, Nanjing Medical University, Nanjing, 211166 China; 2 College of Biomedical Information and Engineering, the First Affiliated Hospital of Hainan Medical University, Hainan Medical University, Haikou, 571199 Hainan China; 3 College of Bioinformatics Science and Technology, Harbin Medical University, Harbin, 150081 Heilongjiang China; 4 Department of Immunology, Nanjing Medical University, Nanjing, 211166 China; 5 Department of Laboratory Medicine, Women and Children’s Hospital of Chongqing Medical University, Chongqing, 401174 China; 6 Baylor College of Medicine, Houston, TX 77030 USA; 7 Shenzhen Institute for Advanced Study, University of Electronic Science and Technology of China, Shenzhen, 518110 Guangdong China; 8 School of Biomedical Sciences and Pharmacy, Faculty of Health and Medicine, the University of Newcastle, University Drive, Callaghan, NSW 2308 Australia; 9 Precision Medicine Research Program, Hunter Medical Research Institute, New Lambton Heights, NSW 2305 Australia

**Keywords:** Single-cell RNA-sequencing (scRNA-seq), Data analysis, Biomedical research, Clinical applications

## Abstract

The application of single-cell RNA sequencing (scRNA-seq) in biomedical research has advanced our understanding of the pathogenesis of disease and provided valuable insights into new diagnostic and therapeutic strategies. With the expansion of capacity for high-throughput scRNA-seq, including clinical samples, the analysis of these huge volumes of data has become a daunting prospect for researchers entering this field. Here, we review the workflow for typical scRNA-seq data analysis, covering raw data processing and quality control, basic data analysis applicable for almost all scRNA-seq data sets, and advanced data analysis that should be tailored to specific scientific questions. While summarizing the current methods for each analysis step, we also provide an online repository of software and wrapped-up scripts to support the implementation. Recommendations and caveats are pointed out for some specific analysis tasks and approaches. We hope this resource will be helpful to researchers engaging with scRNA-seq, in particular for emerging clinical applications.

**Supplementary Information:**

The online version contains supplementary material available at 10.1186/s40779-022-00434-8.

## Background

Complex tissues consist of a variety of cell types that occur in a huge variety of mixtures and states. The functional genomic information contained within each cell is often quite different from the neighboring cell populations and even cells of the same type. This means that the molecular analyses of cell populations in bulk tissues are inherently unreliable and insensitive. The incredible sensitivity and specificity that can be achieved by quantifying molecular alterations at single-cell resolution have led to unprecedented opportunities for uncovering the molecular mechanisms underlying the pathogenesis and progression of the disease [1]. Since its inception, single-cell RNA-sequencing (scRNA-seq) has been shown to be a powerful tool for profiling gene expression in individual cells [2–4], in both physiogenesis [5, 6] and pathogenesis [7–9]. For example, by utilizing scRNA-seq in cancer biology [10, 11], researchers have been able to determine the origin of cancer cells in various tumor types [12, 13]. Moreover, from the treatment and prognosis respect, subpopulations of malignant cells with clinically significant features, such as the poor prognosis in nasopharyngeal carcinoma with dual epithelial–immune characteristics have been discovered [14]. Similarly, strong epithelial-to-mesenchymal transition (EMT) and stemness signatures were observed in metastatic breast cancer cells [15, 16]. With the assistance of scRNA-seq, the quality and validity of organoid systems can also be accurately assessed and systematically evaluated [17–19]. Patient-derived organoid models are currently being applied to the dissection of disease pathology [20] and facilitating drug screening for personalized treatment [21, 22]. Furthermore, distinct cellular states along tumor progress were discovered and drug-resistant cell subsets were identified by joint application of patient-derived organoid and scRNA-seq [23, 24]. In the current coronavirus disease 2019 (COVID-19) pandemic, scRNA-seq accelerates the research for characterizing the molecular basis and, therefore, understanding the pathology of severe acute respiratory syndrome coronavirus 2 (SARS-CoV-2). A variety of scRNA-seq-based studies have revealed the cell subtypes targeted by SARS-CoV-2 [25], profiled gene expression changes in immune cells upon infection [26, 27], quantified the alteration of cell-to-cell interaction between different cell types [26, 28], and provided important resources for the development of potential treatment of COVID-19 [26, 28].

Since the emergence of commercial single-cell platforms, including those offered by 10 × genomics [29, 30] and Singleron [31, 32], scRNA-seq services provided by core facilities of research institutes or third-party companies, are making the technology more accessible, affordable and in some cases a routine technique for biomedical researchers and clinicians [33]. While these service providers typically perform data quality-control and execute basic pipelines for data processing, the high-level data analysis needed for specific research objectives and scientific questions, is not usually available. Thus, most biomedical researchers need to come to grip with the full scope of scRNA-seq data analysis by identifying the most suitable computational tools to dissect their data.

To overcome the barriers in scRNA-seq data analysis, in particular for biomedical studies, this review aims to: 1) summarize the recent advances in algorithm development and benchmarking results for every analysis task in analyzing biomedical scRNA-seq data, and 2) introduce a workflow comprised of recommended software tools that are more appropriate for biomedical applications. The workflow covers basic scRNA-seq data processing, quality control (QC), feature selection, dimensionality reduction, cell clustering and annotation, trajectory inference, cell–cell communications (CCC), transcription factor (TF) active prediction and metabolic analysis. Along with the recommended workflow, we also provide example computational scripts together with the software environment setting, which may facilitate researchers to conduct the data analysis locally. The computational code is available at https://github.com/WXlab-NJMU/scrna-recom. To accommodate upcoming advanced approaches and more application scenarios, we will keep the computational scripts updated.

## General tasks of single-cell RNA-seq data analysis

Typical data analysis steps of scRNA-seq can be generally divided into three stages: raw data processing and QC, basic data analysis applicable for almost all scRNA-seq data sets, and advanced data analysis that should be tailored to specific research scenarios. While basic data analysis steps include data normalization and integration, feature selection, dimensionality reduction, cell clustering, cell type annotation and marker gene identification. The advanced data analysis tasks consist of trajectory inference, CCC analysis, regulon inference and TF activity prediction, and metabolic flux estimation.

### Experimental design

ScRNA-seq experiments need to be carefully designed to optimize the capability in addressing scientific questions [34]. Before starting the data analysis, the following information related to the experiment design needs to be gathered. (1) Species. For biomedical studies and clinical applications, human samples derived from patients are usually collected for sequencing [35–37]. In some cases, to study the underlying molecular mechanisms, mouse and other model organisms are also used [38]. Since the gene names and related data resources are different between humans and other species, it is important to specify the species for data analysis. For simplicity, we will focus on the data derived from human samples. (2) Sample origin. According to the scientific questions and sample accessibility, the sample types can be varied in different studies. For instance, to study solid tumors like hepatocellular carcinoma, tumor biopsies and peritumor samples are collected from patients for a case–control design [39]. Whereas the above design is feasible to some extent, peripheral blood mononuclear cells (PBMCs) are more easily accessible and widely used for scRNA-seq [40, 41]. In addition, cells from patient-derived organoids are often used to study the impact of personal genetic variants on the development of specific organs, which can also be the origin of particular diseases [42, 43]. Knowing the sample origin facilitates particular analysis, such as cell clustering and cell type annotation. (3) Experiment design. To study disease pathogenesis and the effectiveness of particular treatments, a case–control design is mostly adopted, like the tumor-versus-peritumor design [39]. For diseases such as COVID-19, no normal samples can be obtained from the same patients, thus healthy people with matched age and gender serve as a control group [40]. To control possible covariates between the patients and the control groups, the number of individuals in each group needs to be carefully considered [44]. In (prospective) cohort studies, the sample size is usually considerably larger, so that scRNA-seq cannot be applied to every sample from individual donors; in this case, nested case–control studies [45] and sample multiplexing [46] are often applied. In general, data analysis strategies need to be adjusted according to the types of the experiment design.

### Raw data processing

Raw data processing steps include: sequencing read QC, read mapping [47], cell demultiplexing and cell-wise unique molecular identifier (UMI)-count table generation [48]. Whilst standardized data processing pipelines are provided with the release of scRNA-seq platforms, such as Cell Ranger for 10 × Genomics Chromium [49] and CeleScope (https://github.com/singleron-RD/CeleScope) for Singleron’s systems, alternative tools including UMI-tools [48], scPipe [50], zUMIs [51], celseq2 [52], kallisto bustools [53], and scruff [54] can also be used for this procedure. The choice between these pipelines seems less important than the downstream steps according to a recent study benchmarking scRNA-seq analysis [55]. In any case, we would not recommend raw data processing on personal computers, as these pipelines need massive computational resources and are optimized for high-performance computing architectures [56]. Third-party companies usually provide processed data, including UMI count matrices and QC metrics, which enable the researchers to focus on downstream data analysis for addressing scientific questions.

### QC and doublet removal

The purpose of cell QC is to make sure all the ‘cells’ being analyzed are single and intact cells. Damaged cells, dying cells, stressed cells and doublets need to be discarded [57, 58]. In ultrahigh-throughput scRNA-seq, quantitative metrics used for bulk RNA-seq QC, including read mappability, fraction of reads mapped to exonic regions are computed at only the sample/library level, thus cannot be used for cell QC. Instead, the three mostly used metrics for cell QC are: the total UMI count (i.e., count depth), the number of detected genes, and the fraction of mitochondria-derived counts per cell barcode [56, 59]. Cell Ranger [49] and CeleScope (https://github.com/singleron-RD/CeleScope) usually perform a first-round cell QC, which distinguishes potentially authentic cells from background cell barcodes by examining the distribution of count depth in a scRNA-seq library. One caveat is that, when the damaged cells or cell debris take a considerable proportion in the library, the threshold of a minimum count depth for valid cells is hard to be determined. Possible solutions include the consideration of multiple QC metrics at the same time [56], and the application of more sophisticated approaches to rule out background and low-quality cells [60]. Typically, low numbers of detected genes and low count depth indicate damaged cells, whereas a high proportion of mitochondria-derived counts is indicative of dying cells. By contrast, too many detected genes and high count depth can be indicative of doublets [57, 58]. While R packages like Seurat [61–63] and Scater [64] implement functions to facilitate cell QC, the thresholds of the QC metrics are largely dependent on the tissue studied, cell dissociation protocol, library preparation protocol, etc.. Referring to publications with similar experiment designs would help to determine the thresholds, and advanced researchers may also inspect the joint distribution of the QC metrics. Notably, accumulated expression of genes encoding ribosomal proteins is not a typical QC metric, as the variation of ribosomal protein expression can be biologically meaningful [65].

In addition, various sources of contamination need to be considered and controlled during the QC step. For example, libraries derived from PBMCs and solid tissues can be contaminated by red blood cells, and thus cells expressing a high level of hemoglobin genes (e.g., *HBB*) are usually discarded [66, 67]. Another source of contamination is cell-free or ambient RNA, as evidenced by reads mapped back to specific genes in cell-free droplets or wells in high-throughput scRNA-seq [68, 69]. Methods and tools for estimating and removing such contamination have been recently developed, including SoupX [68], DecontX [69], fast correction for ambient RNA (FastCAR) [70] and CellBender [71]. Removal of the background signal caused by ambient RNA in single-cell gene expression improves downstream analyses and biological interpretation [69, 71].

In high-throughput scRNA-seq experiments, it is not uncommon to observe a high rate of doublets, which may reach up to 40% of cell barcodes [72, 73]. For this reason, a filtering step that only considers count depth and the number of detected genes is not adequate, particularly when the cell type composition is complex such that the count depth distribution of singlets is not distinct from that of doublets. Doublets composed of distinct cell types are likely to confound downstream analysis, particularly in cell clustering, differential expression analysis, and trajectory inference [56, 74]. Fortunately, a number of sophisticated approaches have been developed to disentangle these confounding signals [72]. These methods consider the gene expression profiles of individual cell barcodes and report doublet scores as an indicator. The doublet scores are calculated based on either artificial doublets [such as single-cell remover of doublets (Scrublet) [74], doubletCells [75], binary classification based doublet scoring (bcds) [76], DoubletDetection [77], DoubletFinder [78], Solo [73], DoubletDecon [79]] or gene co-expression [such as co-expression based doublet scoring (cxds) [76]]. In a recent study, benchmarking the available computational doublet-detection methods with a comprehensive set of synthetic and real data [72], the tool Doubletfinder [78] was recommended because it achieved both the highest detection accuracy and the best performance in downstream analysis.

### Expression normalization

The variability of total UMI counts per cell depends on a range of both technical and biological parameters [56]. The technical factors relate to the efficiency of RNA capture, reverse transcription, cDNA amplification and sequencing depth, whereas the biological factors mostly relate to cell size and cell cycle phase. Because of this variation, it is almost impossible to obtain the absolute number of RNA molecules unless external spike-in RNA control is added to the sequencing libraries [80, 81]. Like bulk RNA-seq, relative RNA abundance is commonly adopted for comparing gene expression profiles between individual cells; therefore, scRNA-seq data are typically normalized by global-scaling methods with scaling factors developed for bulk RNA-seq [82–84], which suppress partially the technical effects [56]. Popular global-scaling methods for bulk RNA-seq include transcript per million (TPM) [85], upper quartile (UQ) normalization [86], trimmed mean of M values (TMM) normalization [87], and the DESeq normalization method [88], however, are not appropriate for scRNA-seq due the tendency for distortion through zero inflation [81]. Normalization methods tailored for scRNA-seq, including single-cell differential expression (SCDE) [84] and model-based analysis of single-cell transcriptomics (MAST) [82], can specifically model dropout events in differential expression analysis of scRNA-seq data. Another approach, Scran [75], overcomes the issues of scaling factor estimation (affected by too many zero counts) by pooling cells of similar gene expression profiles [89]. Moreover, Census estimates the total number of RNA molecules per cell without spike-in controls and uses these estimates as the scaling factors [90]. While simulation studies carried out by Vallejos et al. [81] suggested Scran’s pooling strategy outperforms compared tools in scaling factor estimation, the TPM-/count depth-scaling method is widely used in practice [91].

Following scaling factor-based normalization, the resulting values are typically added to one pseudo-count and log-transformed [56, 62]. This step is practically useful and statistically sound, as it mitigates the mean–variance relationship in scRNA-seq count data and also reduces the skewness in expression data [56, 64]. Toward better variance stabilization, SCTransform was recently developed by the Seurat team, which applies regularized negative binomial regression for scRNA-seq data normalization and variance stabilization [92].

Some known biological effects, such as cell cycle and cell stress (featured by overexpression of mitochondrial genes), may hinder the characterization of the particular biological signal of interest [56]. Hence, normalizing or correcting expression profiles against known biological may help interpret the data. For instance, correcting the effects of the cell cycle can improve developmental trajectory reconstruction [93, 94]. The procedure accounting for biological effects can be achieved by scoring related biological features (e.g., cell cycle scores [95]), followed by a simple linear regression against the calculated scores as implemented in Seurat [61, 62]. In addition, dedicated tools such as single-cell latent variable model (scLVM)/factorial single-cell latent variable model (f-scLVM) [93, 96] and cell growth correction (cgCorrect) [97] can also be used for this purpose. Of note, correcting biological effects for one particular analysis (e.g., cell differentiation) may unintentionally hinder the signals for another (e.g., cell proliferation) [56]; care should be taken when choosing data normalization strategies for particular analysis tasks.

### Data integration

As mentioned in the ‘Experiment design’ section, biomedical studies usually make case versus control comparisons [39]. Usually, batches of samples obtained from different medical centers or hospitals should be integrated before downstream analysis. For studies using patient-derived organoids, data integration also applies to cells harvested at different time points to depict organoid development [98]. In these cases, one other unwanted technical factor, batch effects, cannot be avoided because cells and library preparation were handled by different persons, at different time points, or with a different batch of reagents [91, 99]. In scRNA-seq, batch effects can be nonlinear, which may not be easily disentangled by state-of-the-art batch correction tools, such as ComBat [100]. Therefore, numerous methods have been recently developed for batch effect correction in scRNA-seq data integration, trying to relieve or remove the effects caused by batch-specific biases while preserving biological variations [56, 99]. The batch effect correction methods can be classified into a few categories: 1) tools developed for bulk expression analysis, including ComBat [100] and limma [101]; 2) approaches based on mutual nearest neighbors (MNN) in high-dimensional gene expression space or its subspace, such as mnnCorrect [102], fastMNN [102], Scanorama [103] and batch balanced k nearest neighbours (BBKNN) [104]; 3) methods that try to align cells with correlated/shared features in dimensionality-reduced spaces, including canonical correlation analysis (CCA) [61, 62], Harmony [105], and linked inference of genomic experimental relationships (LIGER) [106]; and 4) methods based on deep generative models, such as scGen [107]. Besides, depending on the choice of integration anchors, the algorithms can also be sorted into different types, such as genomic features as the anchor and cells as the anchor [108].

Recently, Tran et al. [99] compared 14 batch-effect correction methods available at that time on 10 datasets under 5 different integration scenarios. Among them, Harmony [105], LIGER [106], and CCA implemented in Seurat 3 [62] were recommended according to their overall performance [99]. Together with our experience, it is suggested to perform data integration with Harmony, Seurat3/4-CCA, and LIGER in order. This is because there is no clear winner among the three strategies when dealing with distinct datasets [99]. Harmony runs faster than the other tools, suitable for initial exploration; Seurat3/4-CCA is moderate in mixing cells from different batches, whereas LIGER makes the best efforts in batch mixing, sometimes at the cost of cell type purity. Of note, if one wants to evaluate the effectiveness of batch-effect correction or assess the extent of the batch effects in the data, it can be achieved by comparing clustering or visualization results based on batch-effect corrected analysis and that from directly merging cells derived from multiple samples (e.g., merge function in Seurat), and by computing test metrics such as k-nearest-neighbor batch-effect test (kBET) [91].

### Feature selection

While cell QC removes background cells and problematic cells, the feature section is concerning genes. In the human genome, more than 20,000 genes are annotated, and mapped reads are counted for individual gene loci to yield the UMI count matrix. However, not all the > 20,000 genes are informative in characterizing cell-to-cell heterogeneity or distinguishing cell types/states [56]. Therefore, the term ‘feature selection’ was borrowed from the fields of statistics and machine learning to describe the process of selecting biologically informative genes for downstream analysis. This process is typically unsupervised, meaning that no information related to cell types or other biological processes of interest is needed.

Considering the relatively high noise level in scRNA-seq data, feature selection usually identifies genes with stronger biological variability than technical noise [58]. Since the technical noise largely depends on the mean expression of genes [109], highly variable genes (HVGs) were originally identified by examining the relationship between the coefficient of variation and expression means [58]. Due to its usefulness in reducing technical noise and relieving the computational demand in downstream analysis, such as cell clustering and dimensionality reduction for visualization [110], many other tools for HVG identification were developed and comparatively evaluated [111–113]. Instead of identifying HVGs, alternative feature selection methods consider dropouts and prioritize genes with a higher-than-expected number of observed zeros [114].

The number of genes selected for downstream analysis is theoretically dependent on the complexity of cellular composition in the samples studied. While approaches for HVG identification can determine the number of HVGs at a given significance level, identifying a fixed number of HVGs is becoming popular, and typically the HVG number is between 1000 and 5000 [56]. Studies have shown that downstream analysis is not sensitive to the exact number of HVGs [110, 115]. Notably, some unfavorable covariates such as batch effect may distort HVG identification [82]. Therefore, HVG selection should be performed after correction for the covariates. In the presence of batch effects, feature selection may also be conducted in individual samples before data integration [56].

### Dimensionality reduction and visualization

With 1000–5000 HVGs selected, the dimensionality of the expression data is still high, thus obstructing manual inspection of the dataset, such as visualization, clustering and cell type annotations [116]. To this end, the dimensions of the expression matrixes can be further reduced by dimensionality reduction techniques, which project the cells from a high-dimensional space into a low-dimensional embedding space, and preserve the biological information on cell-to-cell variability [56, 59]. The widely used methods for dimensionality reduction include principal component analysis (PCA) [117], non-negative matrix factorization (NMF) [118], multi-dimensional scaling (MDS) [119], t-distributed stochastic neighbor embedding (t-SNE) [120] and uniform manifold approximation and projection (UMAP) [121].

PCA is a general technique for dimensionality reduction and denoising, and has been widely used in scRNA-seq data analysis [122, 123]. With the linear projection of the original expression matrix to its subspace, PCA gives the principal components (PCs) in order of significance. While the first two or three PCs can be used for visualization, a few more PCs are typically retained for downstream analysis, such as cell clustering and trajectory inference. The number of PCs for retention largely depends on the complexity of the dataset [59], and can be determined by the “elbow” method [56] or the jackstraw permutation-test-based method [95, 124]. Nevertheless, PCA cannot take into account the dropout events in the analysis, which leads to the development of several new methods. Zero-inflated factor analysis (ZIFA) is one of such methods based on factor analysis, which explicitly models the dropout characteristics and outperforms the comparative methods [125]. Similar to PCA, NMF is a linear projection method for dimensionality reduction, and showed robust performance in cell clustering based on scRNA-seq [118].

For visualization, nonlinear dimensionality reduction methods are more suitable, which allow a global nonlinear embedding in a two-/three-dimensional space [126]. MDS is one of the nonlinear dimensionality reduction methods and preserves the distance among the cells in the original space [119]. However, MDS can be not scalable to large-scale scRNA-seq data because calculating the pairwise distances becomes computationally demanding when the number of cells is huge [127]. Emerging evidence suggests t-SNE and UMAP are more suitable for scRNA-seq data, which have been widely used in single-cell analysis for data visualization and cell population identification. However, t-SNE usually suffers from limitations such as slow computation time for large-scale scRNA-seq datasets [128] and global data structure was not preserved [121]. With advantages in the above two respects, UMAP currently becomes the most popular choice for dimensionality reduction. UMAP not only helps visualize the cell clusters but also facilitates annotating the cell clusters. It is worth noting, however, that while UMAP strikes a balance between preserving global data structure and capturing local similarity, the cell-to-cell distance in the resulted space is not preserved. Hence, downstream analysis like clustering and pseudotime inference is typically executed based on the PCA results with several to dozens of PCs.

### Identification of cell subpopulations

**Fig. 1:**
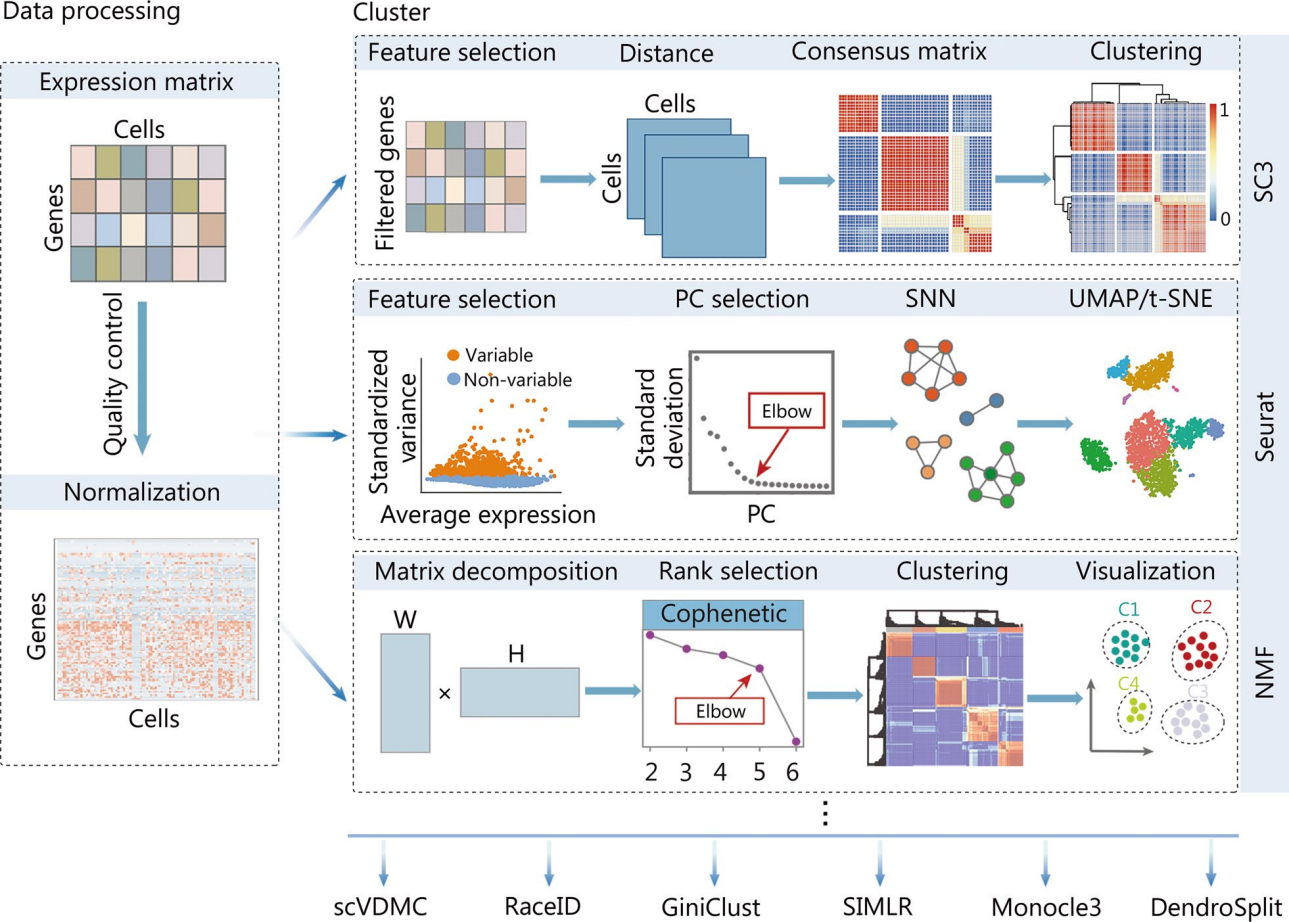
Typical computational strategies and methods for clustering cells using scRNA-seq data. With the processed scRNA-seq data, the SC3 approach, the Seurat clustering implementation based on the community detection method, and the NMF method are popular choices. scRNA-seq single-cell RNA sequencing, SC3 single-cell consensus clustering, NMF non-negative matrix factorization, PC principal component, SNN shared nearest neighbor, scVDMC variance-driven multitask clustering of scRNA-seq data, SIMLR single-cell interpretation via multikernel learning, UMAP uniform manifold approximation and projection, t-SNE t-distributed stochastic neighbor embedding

One of the key applications in single-cell transcriptomics is to determine cell subpopulations based on cell clustering or classification [129, 130]. Due to the high level of noise in the scRNA-seq data, applying dimensionality reduction approaches to scRNA-seq matrix data may facilitate cell clustering. Whilst PCA is commonly used for bulk RNA-seq, the true biological variability of gene expression among cell subpopulations may not be readily distinguished by a small number of PCs. To better account for this variation, NMF was adapted to disentangle subpopulations in single-cell transcriptome data [118, 131], and has been shown to outperform PCA with greater accuracy and robustness (Fig. 1). Likewise, SinNLRR was developed to provide robust clustering of gene expression subspace by non-negative and low-rank representation [132].

State-of-the-art clustering methods, such as the k-means algorithm, have also been applied to scRNA-seq datasets, and based on this application, the single-cell consensus clustering (SC3) approach was developed [133] (Fig. 1). Another category of popularly used methods for cell clustering in scRNA-seq is community detection methods based on a nearest-neighbor network for the cells [134], and was adopted and implemented in the Seurat R package [61] (Fig. 1). Besides, the community has developed a diversity of approaches for cell clustering. For instance, BackSPIN takes advantage of the biclustering technique to avoid unfavorable pairwise comparisons in hierarchical clustering [135], single-cell interpretation via multikernel learning (SIMLR) is based on multi-kernel learning [136], clustering through imputation and dimensionality reduction (CIDR) [137] utilizes imputation to mitigate the impact of dropouts in scRNA-seq, and Single-cell Aggregated Clustering via Mixture Model Ensemble clustering (SAME-clustering) [138] ensembles clustering results from multiple methods. Nevertheless, two independent benchmarking studies have shown that SC3 and the clustering method in Seurat perform similarly to each other and outperform all other comparative methods [139, 140].

Similarity or distance metrics are crucial for clustering cells in scRNA-seq, which can be specific to experiment platforms or particular samples. It has been shown that, compared to unsupervised clustering methods, supervised methods for cell type identification suffered less from batch effects, number of cell types, and imbalance in cell population composition [141]. Mechanistically, the supervised methods rely on a comprehensive reference database with known cell types annotated, based on which a classification model is trained for predicting the cell types in an unannotated dataset [142, 143]. CellAssign [144], scmap [145], single cell recognition (SingleR) [146], characterization of cell types aided by hierarchical classification (CHETAH) [147], and SingleCellNet [148] are methods of this category. Albeit the clear strength of the supervised methods, unsupervised methods are generally better at identifying unknown cell types and have higher computational efficiency [141]. Therefore, the clustering methods implemented in Seurat have the best overall performance, and are suggested as the first choice of cell type identification [141].

Another important issue for single-cell clustering analysis is the detection of rare cell types, which play an important role in complex diseases but have a low abundance. RaceID [129], GiniClust [149], SINCERA [150] and DendroSplit [151] are clustering algorithms specifically designed to identify rare cell types in scRNA-seq data analysis.

### Cell type annotation

**Fig. 2:**
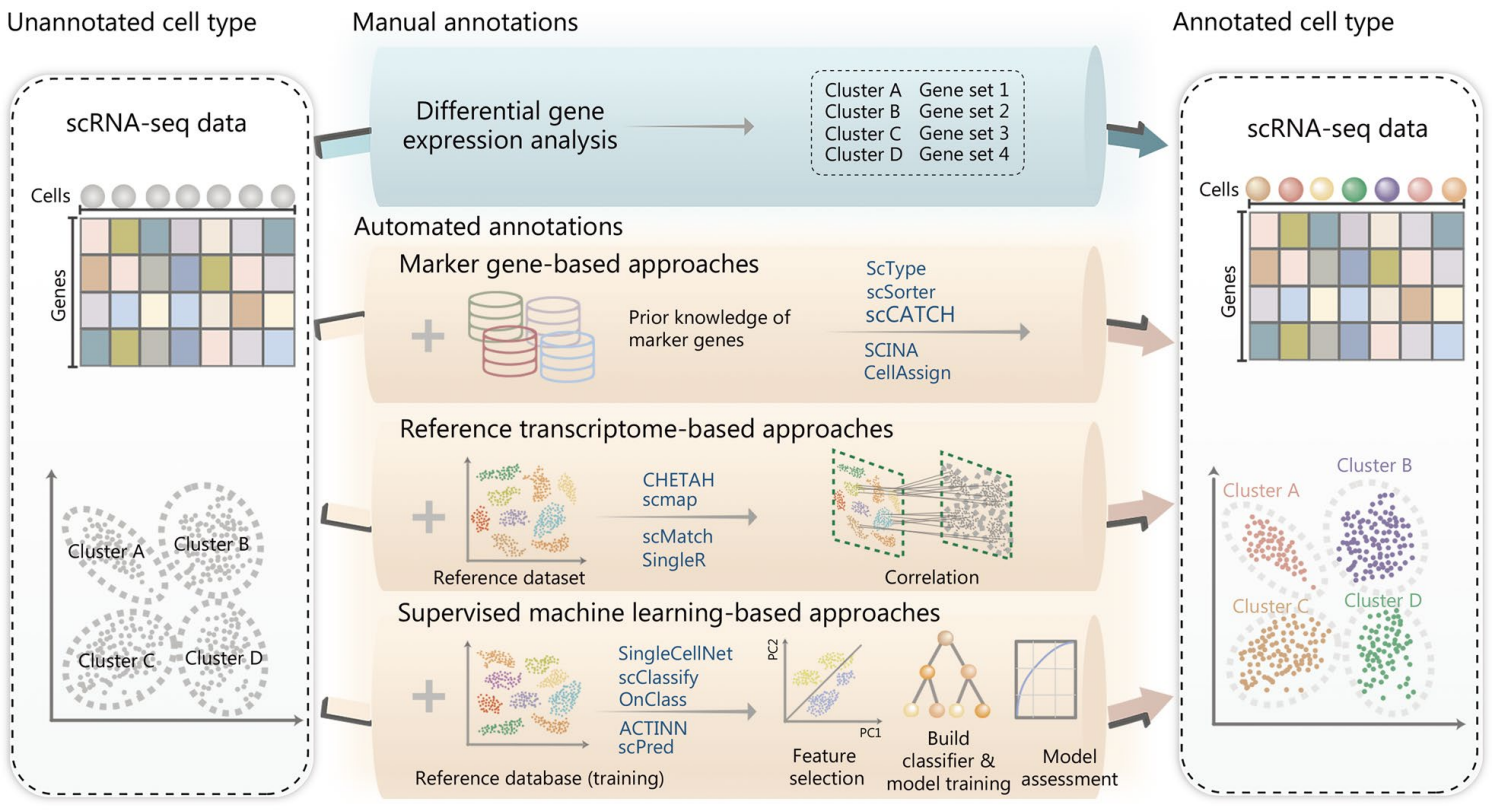
Typical strategies and representative methods for annotating cell subpopulations identified by scRNA-seq. In addition to manual annotation, which is potentially time-consuming and subjective, automated cell type annotation can be mainly sorted into three categories: marker gene-based, reference transcriptome-based, and supervised machine learning-based approaches. The example approach names are listed in the plot. scRNA-seq single-cell RNA sequencing, scCATCH single-cell cluster-based automatic annotation toolkit for cellular heterogeneity, SCINA semi-supervised category identification and assignment, CHETAH characterization of cell types aided by hierarchical classification, SingleR single cell recognition, OnClass ontology-based single cell classification, ACTINN automated cell type identification using neural networks

Assigning cell identities to cell subpopulations, a process known as cell type annotation, is a critical step in scRNA-seq data analysis [152]. Manual annotation of cell types is time-consuming and potentially subjective. Thus, emerging computational tools have been developed for automatic cell type annotation [143, 152]. These computation methods usually can be classified into three main groups (Fig. 2).

The first type is marker gene-based, which relies on the availability of cell type-specific markers in public databases or literature. CellMarker [153] and PanglaoDB [154] are commonly used online resources storing the markers for a large variety of cell types in the tissues of humans and mouse. CellMarker deposits over 13,000 cell markers of about 500 cell types of humans by manually curating over 100,000 published papers [153], and PanglaoDB is a community-curated cell marker compendium, containing 6000 markers for different cell types from over 1000 scRNA-seq experiments [154]. Moreover, the TF-Marker database was developed for providing cell or tissue-specific TFs and related markers for humans [155]. These databases are valuable resources for cell type annotations. Meanwhile, a number of tools have been developed to use the marker genes for cell type annotations, such as ScType [156], scSorter [157], semi-supervised category identification and assignment (SCINA) [158], single-cell cluster-based automatic annotation toolkit for cellular heterogeneity (scCATCH) [159] and CellAssign [144]. Some of these methods apply sophisticated statistical models to make use of the prior knowledge of marker genes. For example, SCINA builds a semi-supervised model to exploit previously identified marker genes with the expectation–maximization (EM) algorithm [158], and CellAssign leverages a probabilistic graphical model to annotate cells into predefined or novel cell types based on prior knowledge of cell-type marker genes, while accounting for batch and sample effects [144].

The second group of methods is reference transcriptome-based, which uses cell type-labeled scRNA-seq datasets as input for cell type annotation, via the search for the best correlation between the queried data and the reference data. Popular tools of this group include CHETAH [147], scmap [145], scMatch [160] and SingleR [146]. The CHETAH algorithm is based on a hierarchical tree built by reference profiles of known cell types, and searches for a cell’s best annotation by stepwise traversing the tree from the root node to a leaf node [147]. By calculating the correlation coefficients between the input cell and two tree branches under consideration based on the 200 most discriminating genes for the two branches, a profile score and confidence score are calculated for selecting tree branches to continue tree traversing. The SingleR approach correlates each unannotated single-cell transcriptome with the reference transcriptomes of known cell types based on HVGs among cell types in the reference data [146]. SingleR assigns cell identity in an iterative manner, and in each iteration the reference set is reduced to refine the assignment. Notably, the comprehensiveness of the reference transcriptomics data is critical for this group of methods. The reference data from Blueprint [161], Encode [162] and the Human Primary Cell Atlas [163] are commonly used.

Lastly, the third group leverages supervised machine learning-based approaches, where classifiers trained by a labeled reference are then applied to predict cell types of unannotated cells. For instance, SingleCellNet uses multi-class random forest classifiers [148], automated cell type identification using neural networks (ACTINN) uses artificial neural networks [164], scPred uses support vector machine (SVM) [165], and scClassify uses ensemble learning [166] for cell type annotation. Furthermore, ontology-based single cell classification (OnClass) may also accurately annotate cell types absent in the training dataset, through identifying the nearest cell type in low-dimensional embeddings resulting from the Cell Ontology and the unannotated cells [167].

Automated methods for cell type annotation have been applied in a broad range of biomedical studies, including cancer research. However, a recent benchmarking study has demonstrated that every computational method possesses specific advantages over the others under different scenarios [142], making it however difficult for clinical users to select the appropriate tools. Integrating the annotation results from multiple tools may be a solution to the above issue, and probably achieve more accurate cell types annotation. Therefore, ImmCluster has been developed recently for immune cell clustering and annotation, integrating seven reference-based and four marker gene-based computational methods, supported by manually curated marker gene sets [168]. Comparative studies have shown that ImmCluster provides more accurate and stable cell type annotation than individual methods [168].

### Marker gene identification

Marker genes of a particular cell cluster or cell type are an important resource for characterizing its function. In reverse, as shown above, marker genes can also be used for cell type annotation. The typical methods to identify cell cluster/type-specific genes are those to identify differentially expressed genes (DEGs) among the clusters based on statistical tests. For example, the scRNA-seq analysis pipelines Seurat [169] and SINCERA [150] use the nonparametric Wilcoxon’s rank-sum test to identify highly expressed genes of specific cell types. It has been shown that Wilcoxon’s rank-sum test is of low false positive rates than dedicated methods for sequencing-based DEG analysis [e.g., DESeq2 [170] and empirical analysis of digital gene expression (DGE) in R (edgeR) [171] when the sample size is large [172]]. In addition, the nonparametric Kruskal–Wallis test was adopted in SC3 [133] for comparisons of more than two groups of cells. Considering dropouts in scRNA-seq and differences in gene expression distribution between cell types or status, many other methods have been developed for marker genes identification, such as MAST [82], SCDE [84], and DEsingle [173].

There is one more category of methods, which identify cell-specific genes simultaneously with the process of cell clustering rather than a step thereafter. As introduced in the earlier section, BackSPIN is based on a biclustering approach [135], which clusters highly expressed genes together when clustering cells. Similarly, iterative clustering and guide-gene selection (ICGS) first identifies guide genes by pairwise correlation of expressed genes, and then performs iterative clustering with the guide genes [174]. Moreover, DendroSplit considers marker genes’ significance level in identifying sub-clusters [151]. Finally, statistically modeling the distribution of gene expression across individual cells, methods like variance-driven multitask clustering of scRNA-seq data (scVDMC) [175], BPSC [176] and bias-corrected sequencing analysis (BCseq) [177] have been developed to improve both cell subtype identification and differential expression analysis.

Regarding the best choice of DEG tools in scRNA-seq, a recent study compared 36 approaches and found fundamental differences between the methods compared [178]. It has been pointed out that prefiltering of lowly expressed genes may help DEG analysis, and the methods used for bulk RNA-seq analysis in general have comparable performance to those specifically developed for scRNA-seq. Overall, the nonparametric Wilcoxon’s rank-sum test ranks high in most application scenarios, except for complex experimental designs.

### Functional enrichment analysis

To facilitate the interpretation and organization of marker genes identified in each cell type, functional enrichment analysis is commonly performed. Computational methods developed for bulk transcriptomics can be easily applied to this analysis, such as Database for Annotation, Visualization, and Integrated Discovery (DAVID) [179]. This kind of analysis requires a hard cutoff on statistical significance to define the marker genes; in contrast, the widely-used gene set enrichment analysis (GSEA) is a cutoff-free approach [180, 181]. GSEA begins with ordering genes based on differential expression statistics between cell populations of interest, followed by statistically assessing if a functionally meaningful gene set or pathway is significantly overrepresented toward the top or bottom of the ranked list. To facilitate GSEA analysis, Molecular Signatures Database (MSigDB) provides a series of annotated gene sets, including pathways and hallmark gene signatures [182].

Besides the above scenarios where the functional annotation is performed based on marker genes or differential expression between two groups of cells, this analysis can also be carried out at the single-cell level. Single sample GSEA (ssGSEA) and gene set variation analysis (GSVA) [183], which are analogues to GSEA and designed for enrichment analysis of single bulk samples, have now been widely used in scRNA-seq to compute signature scores [184, 185]. Besides, accounting for its characteristics in scRNA-seq, more specific tools including Vision [186], Pagoda2 [187], AUCell [188], single-cell signature explorer (SCSE) [189] and jointly assessing signature mean and inferring enrichment (JASMINE) [190] have been proposed, and in general more suitable for signature scoring in scRNA-seq [190]. In addition, these signature-scoring methods can also be used for pathway activity inference [185].

### Trajectory inference and RNA velocity

In addition to the cell-to-cell heterogeneity that can be captured by scRNA-seq, the dynamics of transcriptomes may also reflect the developmental trajectory or cell state transitions. Trajectory inference [191], pseudo-time estimation [192], and RNA velocity modeling [193] are all helpful to reveal molecular characteristics and regulatory mechanisms during cell differentiation or activation.

Trajectory inference is a popular research field in the past years, with approximately a hundred computational tools developed [191], facilitating studies in developmental biology, as well as cancer development and immune response status alterations. Furthermore, applying this category of methods may also facilitate the objective identification of new cell types [194], and the inference of regulatory networks during the development or status transition [188]. According to the types of trajectories, the trajectory inference methods can also be classified into different categories, including linear methods [e.g., SCORPIUS [195], tools for single cell analysis (TSCAN) [196], Wanderlust [197]], bifurcating methods [e.g., diffusion pseudotime (DPT) [198], Wishbone [199]], multifurcation methods [e.g., FateID [200], STEMNET [201], mixtures of factor analysers (MFA) [202]], tree methods (e.g., Slingshot [203], scTite [204], Monocle [205]), and graph methods [e.g., partition-based graph abstraction (PAGA) [206], rare cell type identification (RaceID) [129], selective locally linear inference of cellular expression relationships (SLICER) [207]]. Currently, the trajectory inference methods are maturing, particularly for the linear and bifurcating methods [191]. Based on a recent benchmarking study, guidelines for practical applications are given so that biomedical researchers can choose the appropriate methods according to prior knowledge on the expected topology in the data [191]; otherwise, PAGA, Monocle, RaceID, and Slingshot are recommended for an initial investigation.

Per existing biological knowledge on the starting point of inferred developmental or transition trajectory, cells along the trajectory can be ordered in a pseudo-temporal order. If there are bifurcation, multifurcation, or tree structures in the trajectory, multiple routes should be applied to go through tree branches separately. In this manner, it is easy to investigate gene expression dynamics along the pseudo time. Methods have been developed to conduct the trajectory-/pseudotime-based differential expression analysis [208, 209], which may reveal the dynamic regulation of lineage/status specification.

An alternative way to capture transcriptome dynamics is to use RNA velocity, which is based on the relationship between matured and unmatured transcripts (i.e., with unspliced introns) in the same cell. If there are relatively more unspliced transcripts in a cell, the gene is under upregulation, and vice versa. Jointly quantifying the ratio between matured and unmatured transcripts, and the gene expression changes during status changes, the direction of cell transition can be thus determined [192]. This rationale has been realized in the first RNA velocity method Velocyto [210], and improved in the follow-up method scVelo, where a likelihood-based dynamical model was adopted [211]. Furthermore, recently developed methods [212, 213] have combined RNA velocity with trajectory inference, resulting in directed trajectory inference independent of prior knowledge. For instance, CellRank takes advantage of both the robustness of trajectory inference and the directional information from RNA velocity, enabling the detection of previously unknown trajectories and cell states [212]. CellPath is another method integrating single-cell gene expression dynamics and RNA velocity information for trajectory inference [213].

### Cell–cell communications

CCC events play important roles in organism development and homeostasis, as well as disease generation and progression. For example, tumor microenvironments are complex ecosystems composed of tumor cells, stromal cells and a variety of immune cells, such that abnormal or disrupted communication among these cells may promote tumor growth. To this end, various computational tools have been developed to infer CCC using scRNA-seq data [214]. The communication between cells commonly depends on ligand-receptor (LR) interactions, which are usually quantified by LR co-expression.

**Fig. 3:**
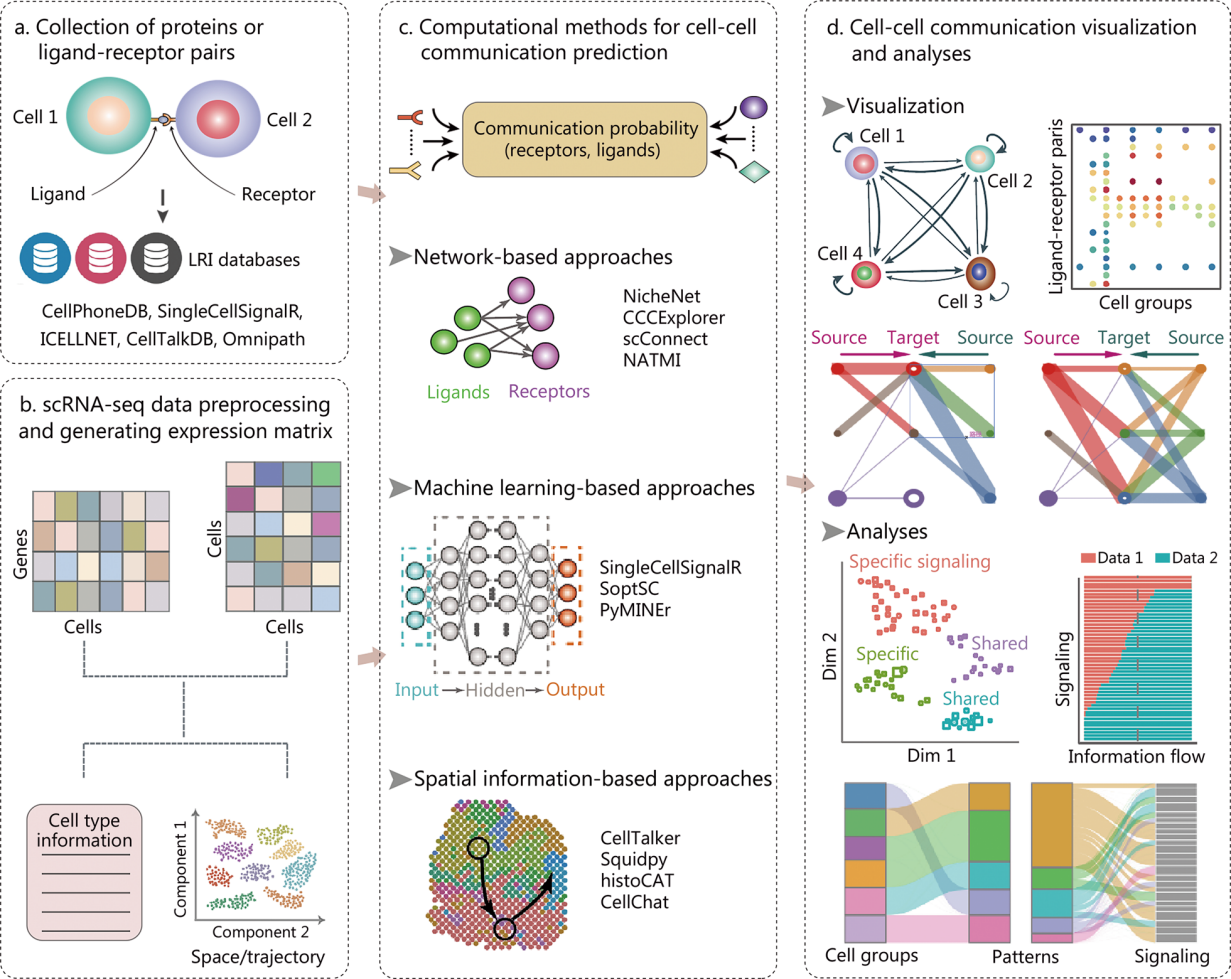
The data resources, computational pipelines, and visualization methods used for cell–cell communication (CCC) inference with scRNA-seq data. Typical analysis steps include the collection of ligand-receptor pairs (**a**), cell clustering and annotation in scRNA-seq (**b**), computational prediction of CCC (**c**), followed by results visualization and downstream analysis (**d**). The CCC inference tools can be categorized into three main classes: network-based, machine learning-based and spatial information-based approaches. LRI ligand-receptor interaction, scRNA-seq single-cell RNA sequencing, CCCExplorer cell–cell communication explorer, NATMI network analysis toolkit for multicellular interactions, histoCAT histology topography cytometry analysis toolbox, SoptSC similarity matrix-based optimization for single-cell data analysis, PyMINEr Python maximal information network exploration resource, Squidpy spatial quantification of molecular data in Python

To facilitate the above investigation, known ligand-receptor interactions (LRIs) have been manually curated and deposited in databases (Fig. 3a). To date, there are quite a few LRI databases, including CellPhoneDB [215], ICELLNET [216], CellTalkDB [217], SingleCellSignalR [218] and Omnipath [219]. The last updated CellPhoneDB (version 4) includes nearly 2000 high-confidence interactions between ligand and receptor proteins, as well as heteromeric protein complexes [215, 220]. CellTalkDB is another comprehensive LRI database in humans and mouse, including 3398 human LR pairs and 2033 mouse LR pairs [217]. Meanwhile, scRNA-seq data are processed using methods mentioned previously for cell clustering and annotation (Fig. 3b). Integrating the annotated scRNA-seq data with known LRIs, sample-specific LR scores are typically calculated, quantifying the interaction potential. Based on LR co-expression, there are a few categories of LR scoring functions [221], including expression thresholding, expression correlation, expression product, and a combination of differential expression [222]. For example, Camp et al. [223] only considered LR pairings if the expression values of both the ligand and receptor were above a certain threshold [log_2_(FPKM) ≥ 5]. By contrast, the method SingleCellSignalR is based on the product of LR gene expression levels [218].

Recently, computational methods for predicting CCC based on scRNA-seq data have been continuously developed [221]. The CCC inference tools can be categorized into three main classes according to their special features (Fig. 3c), that is network-based, machine learning-based and spatial information-based approaches [221]. Network-based approaches, including NicheNet [224], cell–cell communication explorer (CCCExplorer) [225], scConnect [226] and network analysis toolkit for multicellular interactions (NATMI) [227], leverage the connection network between genes to predict CCC. For instance, NicheNet integrates single-cell expression data with prior knowledge of signaling pathways and gene regulatory networks [224], featured by the application of personalized PageRank algorithm, which was used to calculate ligand–target regulatory potential scores [228]. Various types of machine learning algorithms are adopted in the machine learning-based approaches, such as SingleCellSignalR [218], similarity matrix-based optimization for single-cell data analysis (SoptSC) [229] and Python maximal information network exploration resource (PyMINEr) [230]. Besides, reference component analysis (RCA)-CCA [231], linear regression [232] and decision tree classifiers [233] were also used for CCC prediction. Cell localization in space or spatial proximity between cells is the prerequisite of CCC; hence, accounting for spatial information would improve the accuracy of CCC inference. With the rapid development of spatial transcriptomics, many CCC inference approaches integrate scRNA-seq data with spatial transcriptomic and/or image data for identifying CCC. CellTalker scored communication among cell types by counting the number of LRIs, which was then assessed by spatial proximity between cells using image data [234]. In addition, spatial quantification of molecular data in Python (Squidpy) [235] and histology topography cytometry analysis toolbox (histoCAT) [236] provide analysis frameworks for spatial omics data, where intercellular communication can be investigated through cellular proximity or neighborhood analysis. Moreover, the authors of CellChat take the spatial information as the gold standard to evaluate different CCC inference approaches, and showed that CellChat performs better at predicting stronger interactions [237]. Finally, the inference results are usually visualized by heatmap, circus plot, Sankey plot and bubble plot (Fig. 3d).

The emerging computational methods for identifying CCC have improved our understanding of the microenvironment for disease development. However, all the methods depend on prior knowledge of LRIs and statistical or machine learning models to predict potential CCC events. Alternatively choosing LRI resources and prediction approaches may result in different results, yet the impact of the choice on the results is largely unknown. To address this issue, one recent study systematically compared 16 resources and 7 methods for CCC inference, as well as the consensus of the compared methods [214]. The comparison demonstrated that different LRI resources covered a varying fraction of the collective prior knowledge, and the predicted CCC were largely inconsistent with each other, suggesting the need for continued efforts to improve CCC-inference resources and tools.

### Regulon inference and TF activity prediction

**Fig. 4:**
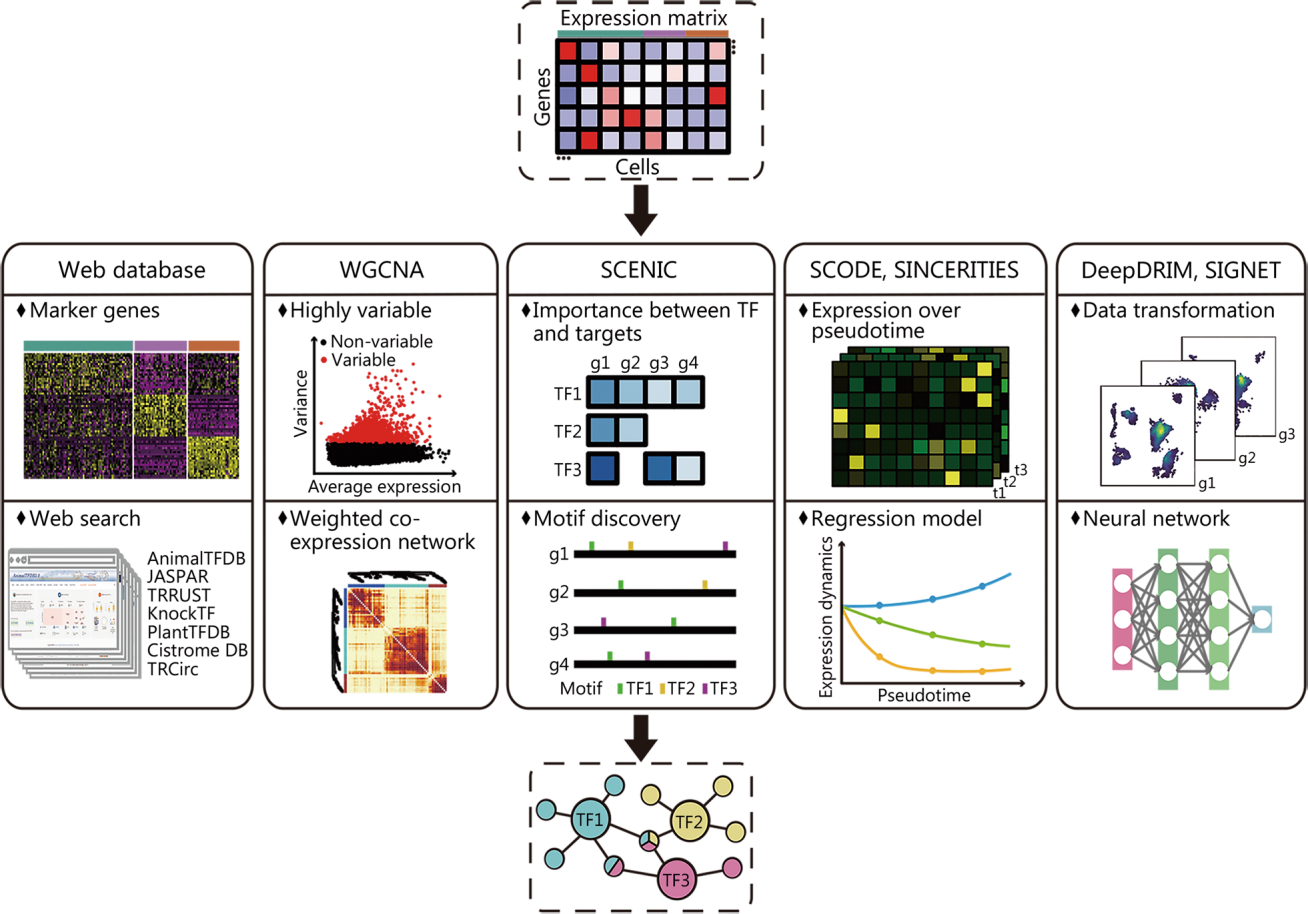
Different strategies and approaches developed for regulon inference and TF activity prediction with scRNA-seq. To achieve regulon and TF activity prediction, the TF databases and TF-target databases are important resources, and the computational strategies include co-expression gene module identification, dynamic and stochastic modeling of TF versus target expression changes, and application of machine learning approaches. TF transcription factor, scRNA-seq single-cell RNA sequencing, AnimalTFDB Animal Transcription Factor DataBase, Cistrome DB Cistrome Data Browser, WGCNA weighted gene co-expression network analysis, SCENIC single cell regulatory network information and clustering, TRRUST transcriptional regulatory relationships unravelled by sentence-based text-mining

TFs play essential roles in gene expression regulation, and are involved in various physiological and pathological processes of humans [238]. It has been realized in scRNA-seq to identify co-expression modules that were directly regulated by TFs of interest, and these modules were defined as regulons [188]. Therefore, it has been made possible to chart the cell type-specific regulons and to reconstruct regulation-based regulatory networks in individual cells (Fig. 4).

One important resource in recognizing regulons is the TF-target databases. The Animal Transcription Factor DataBase (AnimalTFDB) [239], JASPAR [240], transcriptional regulatory relationships unravelled by sentence-based text-mining (TRRUST) [241], KnockTF [242], and Cistrome Data Browser (Cistrome DB) [243] are widely applied TF annotation databases, covering most human and mouse TFs. Based on these databases, a simple way to build cell type-specific transcriptional regulatory networks is to identify up-regulated TFs and/or differentially expressed TF-target genes. For instance, a recent scRNA-seq study identified differentially expressed TFs based on AnimalTFDB TF annotation, and revealed that the reactivation of TFs expressed in fetal epithelium may be the cause of Crohn’s disease [244].

Integrating single-cell gene expression and the comprehensive TF-target information, there have been many methods developed for inferring regulons and TF activity. Coexpression analysis, such as weighted gene co-expression network analysis (WGCNA) [245], has been widely used in bulk samples to detect gene modules that likely are regulated by the same TF(s). Recently, this approach has also been applied to scRNA-seq data, to discover, for example, the gene modules whose expression changed significantly over the course of HIV infection [246]. The single cell regulatory network information and clustering (SCENIC) method is the earliest method for regulon inference based on scRNA-seq data [188], and has now been used to study regulatory networks of many diseases such as cancer and COVID-19 [247, 248]. In SCENIC, co-expression modules between TFs and their target genes are first inferred with machine learning methods such as random forest regression, followed by regulon identification through TF’s binding motif analysis, and only their direct targets in the co-expression modules are kept to form the regulons. Finally, binarized scores are calculated to indicate TF’s activity in each cell. The other methods, including SCODE [249] and SINCERITIES [250], take advantage of the pseudo-temporal information reconstructed in scRNA-seq and infer TF-target regulatory networks based on ordinary differential equations or stochastic differential equation models. Moreover, machine learning techniques have also been applied for transcriptional regulation analysis. For example, while SIGNET [251] adopts multiple-layer perceptron bagging to identify regulons, DeepDRIM [252] utilizes supervised deep neural network to reconstruct gene regulatory networks. In particular, DeepDRIM is shown to be tolerant to dropout events in scRNA-seq and identify distinct regulatory networks of B cells in COVID-19 patients with mild and severe symptoms.

Despite many methods developed for gene regulation analysis based on scRNA-seq, a rigorous judgment on the inferred results needs to be made, due to the complexity of transcriptional regulation and the insufficient information provided by scRNA-seq data. Performing validation experiments may make the inferred results more solid [253, 254].

### Metabolic analysis

Metabolism is at the core of all biological processes, and metabolic dysregulation is a hallmark of many diseases including cancer, diabetes, and cardiovascular disease [255]. Although single-cell metabolomics technologies are under rapid development, they are now too premature for large-scale applications [256]. Instead, metabolic analysis based on single-cell transcriptomics is a promising alternative approach. For example, researchers may use scRNA-seq to monitor the gene expression changes of key metabolic genes under different treatments [257] or during important physiological/pathological processes [258].

**Fig. 5:**
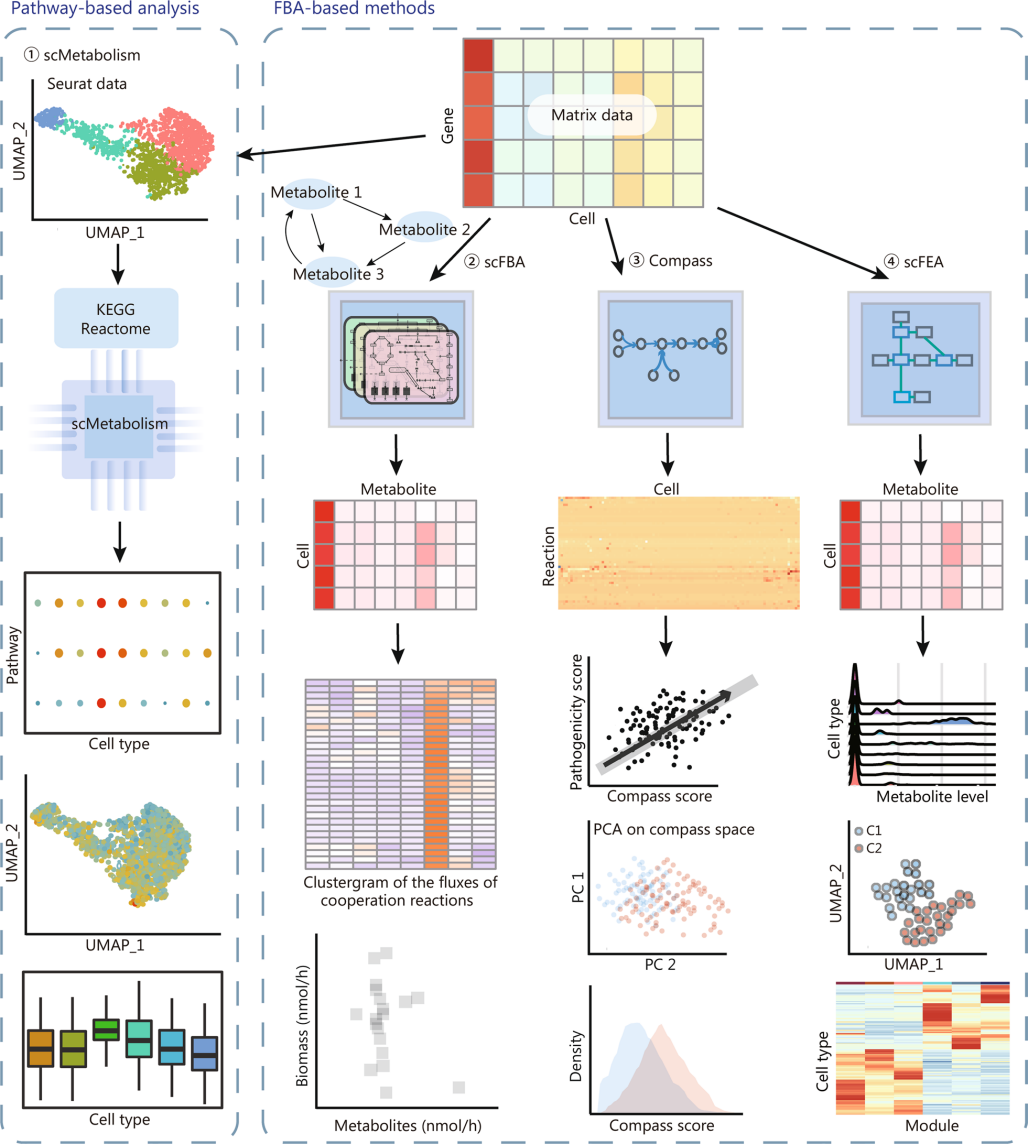
Two main types of metabolic analysis within scRNA-seq: pathway-based functional enrichment analysis and flux balance analysis of metabolic flow. While the former makes use of standard functional enrichment analysis, and the latter utilizes constraint-based mathematical models to systematically simulate metabolism in metabolic networks. Methods including scFBA, Compass, and scFEA employed different implementation strategies for flux balance analysis of metabolic flow. FBA flux balance analysis, KEGG Kyoto Encyclopedia of Genes and Genomes, UMAP uniform manifold approximation and projection, scRNA-seq single-cell RNA sequencing, scFBA single-cell flux balance analysis, scFEA single-cell flux estimation analysis, PCA principal component analysis

The computational tools for scRNA-seq-based metabolic analysis can be classified into two major categories: pathway-based analysis and flux balance analysis (FBA)-based methods [256] (Fig. 5). For the first category, the standard functional enrichment analysis approaches are generally used (refer to the subsection entitled Functional enrichment analysis). In particular, the R package scMetabolism provides an integrated framework for quantitative analysis of metabolic pathway activity in scRNA-seq, with the ability to account for dropouts, and compatible with multiple tools designed for single-cell functional enrichment analysis [259], including ssGSEA [183, 184], Vision [186], and AUCell [188].

The other category is the FBA-based methods, where constraint-based mathematical models are utilized to systematically simulate metabolism in reconstructed metabolic networks [260]. The reconstruction of metabolic networks is usually based on curated databases, such as Kyoto Encyclopedia of Genes and Genomes (KEGG) [261] and Reactome [262]; thereafter, FBA computes static metabolic fluxes in the system with constraints on the input and output fluxes satisfied [263]. Expression levels of individual enzymes in single cells may not directly affect metabolic fluxes in the networks, because they are mostly dependent on the network topology and constraints [256]. To our knowledge, single-cell flux balance analysis (scFBA) was the first computational tool that combines scRNA-seq data and FBA to estimate single-cell fluxomes [264]. Later, Compass [265] and single-cell flux estimation analysis (scFEA) [255] were proposed. Compass is based on Recon2’s reconstruction of human metabolism [266] and solves constraint-based optimization problems with linear programming, to score the potential activity of each metabolic reaction in individual cells [265]. By contrast, scFEA introduces a probabilistic model to consider the flux balance constraints, a multiplayer neural network to model the nonlinearity of flux changes and enzymatic gene expression changes, and a graph neural network to solve the optimization problem [255]. The analysis result by scFEA enables a variety of biologically meaningful downstream analysis, such as cell–cell metabolic communications.

## A collected resource for scRNA-seq data analysis with biomedical applications

With the above overview of the analysis steps and tools for scRNA-seq data, this review may help biomedical researchers to design the data processing and analysis frameworks. However, it would still be challenging for researchers without a bioinformatics background to implement the analysis tasks for their data. For instance, scRNA-seq data analysis requires the installation of specific software tools and running through the scripts written in programming languages such as R and Python. To this end, we collected a range of widely-used software tools in scRNA-seq, and provided practical guidance for installing and running through the analysis with simple commands. The software collection, practical examples, brief description of the analysis results are available at https://github.com/WXlab-NJMU/scrna-recom. Notably, due to time and space constraints, we are unable to incorporate all popular tools into the analysis pipelines on the GitHub site; however, we provide a list of currently available tools with accessible links for users’ convenience (Additional file 1: Table S1). We are also open to suggestions from the community and will adjust the pipelines accordingly. Currently, there are still a few research domains in scRNA-seq data analysis that are under positive development, we will keep updating related software and adjusting the scripts to implement the favorable progress made in these research domains.

## Discussion

Focusing on single-cell transcriptomics, we have reviewed almost all respects of typical analysis of scRNA-seq data, ranging from QC, basic data processing, to high-level analysis including trajectory inference, CCC estimation and metabolic analysis. To facilitate researchers conducting the analysis on their data, we have constructed an online software/script repertoire for these analysis steps, and will keep it updated to cover more research scenarios. We also offer a step-by-step command line interface (CLI) for wrapping up the R and Python scripts for scRNA-seq analysis. The step-wise commands can be flexibly combined and tailored for specific applications due to the diversity on scientific questions and experimental design. Moreover, incorporating cutting-edge technologies, the analysis steps reviewed above may not cover every specifically required task. Indeed, additional analysis pipeline (https://github.com/WXlab-NJMU/scPolylox) was necessary to process the scRNA-seq data for identifying *Polylox* transcript variants in lineage tracing [267].

In this review, we did not mention the task for gene expression imputation aiming to alleviate the impact of the well-known dropout issue in scRNA-seq [268]. This is because all the analyses reviewed in this article can be carried out without data imputation, and moreover one comparative study reported that the imputation results did not improve downstream analysis compared to no imputation [269]. Nevertheless, expression data imputation may help when the expression diversity of important genes or gene pairs needs to be investigated [270]. Additionally, the data integration step for removing the effect of covariants can also be optional. For instance, in a complex experimental design where tumor tissues and peritumor tissues are collected from liver cancer patients of different cancer subtypes, the strategies to integrate the datasets may be different depending on whether the common feature of the liver cancer or the subtype-specific feature is interesting.

Previous research has classified the downstream scRNA-seq data analysis methods into cell-level and gene-level analysis [56], which is intuitive and helpful for understanding. While cell-level analysis is typically concerned with the cell composition of given tissues or samples, gene-level analysis focuses on gene expression differences and heterogeneity. As a result, cell clustering for subpopulation identification, trajectory analysis, and CCC inference are examples of cell-level analysis, whereas differential expression, functional enrichment analysis, regulon inference, and metabolic flux analysis are primarily concerned with gene-level information. In contrast to bulk RNA-seq, single-cell RNA-seq allows for cell-level analysis with unprecedented accuracy and throughput, which in turn inspires a few types of gene-level analysis, such as marker gene identification and gene expression dynamics along inferred trajectories.

One more important point in scRNA-seq data analysis is data presentation and interpretation. Although there are no standard protocols for presenting and interpreting the analysis results, these procedures directly link the data with scientific conclusions. In particular, choosing the most appropriate plots would make the message conveyed more straightforwardly. For instance, if one wants to compare the expression levels of a particular gene between tumor and peritumor samples, violin plots showing the two distributions of the expression levels would be more appropriate than t-SNE or UMAP visualizing individual cells with color scales indicating the expression levels. Moreover, using t-SNE or UMAP visualization to compare the composition of cell origins (e.g., from tumor samples or peritumor samples) in a cell subtype of interest might be misleading, although it is more intuitive. This is because massive cells are usually profiled in a scRNA-seq experiment, and consequently cell points can be buried by some others in the two-dimensional visualization. Other types of plots that directly and more quantitatively demonstrate the composition would be more suitable.

Many other aspects of scRNA-seq data analysis are advancing rapidly. ScAPAtrap [271], Sierra [272], dynamic analysis of alternative polyadenylation (APA) from single-cell RNA-seq (scDaPars) [273], SCAPTURE [274], and single cell alternative polyadenylation using expectation–maximization (SCAPE) [275], for example, take advantage of the fact that sequencing reads in 3’ tag-based scRNA-seq are distributed near the polyadentation sites of individual transcripts to analyze alternative polyadentation and differential usage of 3’UTR isoforms between cells or cell types. Alternative UTR isoform usage is an important post-transcriptional regulatory mechanism in many physiological and pathological processes, affecting the rate of RNA degradation and the status of translation [276, 277]. Currently, many research groups have been combining scRNA-seq with long-read sequencing technologies to enable high-confidence isoform profiling at the single-cell level [278–280]. Such studies have paved the way for the examination of alternative splicing and transcript fusions between cells and/or cell types, as well as during the progression of diseases [278].

In addition to gene expression regulation by TFs, trans-factors like RNA binding proteins (RBPs) and microRNAs typically bind to the 3’UTR of genes to modulate RNA stability, which also contributes to cellular RNA concentration. Based on collections of RBP and microRNA target genes [281, 282], RBP and microRNA regulons can be investigated similarly to the TF regulons [283] in scRNA-seq. In fact, this kind of co-expression module-based analysis can be extended to the examination of cellular signaling pathway activities. Furthermore, in conjunction with CCC inference [214] and ligand–target regulatory potential scores [224], the activation of certain signaling pathways may also be inferred using scRNA-seq data.

Very recently, Live-seq has been developed to convert scRNA-seq from an end-point type assay to a temporal analysis workflow, by keeping cells alive while extracting RNA from individual cells [284]. It is anticipated that Live-seq will address a number of additional biological questions beyond scRNA-seq. In addition, other sequencing-based single-cell profiling technologies are under rapid development. Aiming at better understanding the dysregulation of altered gene expression in diseases conditions, single-cell assay for transposase-accessible chromatin using sequencing (ATAC-seq) [285], single-cell DNA methylation profiling [286], and single-cell Hi-C [287] are all useful to dissect the underlying regulatory mechanisms from different angles at the single-cell resolution. Algorithms have also been developed to integrate these multimodal single-cell data [63], capable of better resolving cell states and defining novel cell subtypes. Moreover, single-cell multi-omics approaches enable simultaneously profiling a couple of omics in identical cells [288], providing information on both regulatory elements and consequential gene expression levels for individual cells. The datasets generated by these technologies may help biomedical researchers to discover disease-specific regulatory programs, possibly in the subset of certain cell types [289]. Furthermore, although still in the developmental stage, spatial transcriptomics is a promising technique for considering the cellular context in characterizing molecular features of a particular cell [290]. With ever-increasing resolution in spatial transcriptomics, we anticipate gaining more in-depth knowledge in analyzing cell microenvironment and cell–cell interactions in health and disease. Collectively, with technologies continuously advancing, especially those that resolve molecular properties and interactions at the single-cell resolution, we will be able to better understand the pathogenesis of a variety of diseases and enable personalized therapies in the near future.

## Supplementary Information

**Additional file 1. Table S1**: Tools for analyzing single-cell RNA-seq data, with references and links.

## Data Availability

The online repository of software and wrapped-up command line interface (CLI) is available at https://github.com/WXlab-NJMU/scrna-recom.

## Abbreviations

AnimalTFDB: Animal Transcription Factor DataBase
ACTINN: Automated cell type identification using neural networks
APA: Alternative polyadenylation
ATAC-seq: Assay for transposase-accessible chromatin using sequencing
BBKNN: Batch balanced k nearest neighbours
bcds: Binary classification based doublet scoring
Bcseq: Bias-corrected sequencing analysis
CCA: Canonical correlation analysis
CgCorrect: Cell growth correction
CCC: Cell–cell communications
CHETAH: Characterization of cell types aided by hierarchical classification
Cistrome DB: Cistrome Data Browser
CIDR: Clustering through imputation and dimensionality reduction
CLI: Command line interface
COVID-19: Coronavirus disease 2019
cxds: Co-expression based doublet scoring
DAVID: Database for Annotation, Visualization, and Integrated Discovery
DEGs: Differentially expressed genes
DGE: Digital gene expression
DPT: Diffusion pseudotime
EMT: Epithelial-to-mesenchymal transition
EM: Expectation-maximization
f-scLVM: Factorial single-cell latent variable model
FastCAR: Fast correction for ambient RNA
FBA: Flux balance analysis
GSEA: Gene set enrichment analysis
GSVA: Gene set variation analysis
HVGs: Highly variable genes
HistoCAT: Histology topography cytometry analysis toolbox
ICGS: Iterative clustering and guide-gene selection
JASMINE: Jointly assessing signature mean and inferring enrichment
kBET: k-nearest-neighbor batch-effect test
KEGG: Kyoto Encyclopedia of Genes and Genomes
LR: Ligand-receptor
LRI: Ligand-receptor interaction
LIGER: Linked inference of genomic experimental relationships
MAST: Model-based analysis of single-cell transcriptomics
MSigDB: Molecular Signatures Database
MDS: Multi-dimensional scaling
MFA: Mixtures of factor analysers
MNN: Mutual nearest neighbors
NATMI: Network analysis toolkit for multicellular interactions
NMF: Non-negative matrix factorization
OnClass: Ontology-based single cell classification
PAGA: Partition-based graph abstraction
PBMCs: Peripheral blood mononuclear cells
PCA: Principal component analysis
PCs: Principal components
PyMINEr: Python maximal information network exploration resource
QC: Quality control
RaceID: Rare cell type identification
RCA: Reference component analysis
RBPs: RNA binding proteins
SCINA: Semi-supervised category identification and assignment
SARS-CoV-2: Severe acute respiratory syndrome coronavirus 2
SCAPE: Single cell alternative polyadenylation using expectation–maximization
SingleR: Single cell recognition
SCENIC: Single cell regulatory network information and clustering
SCSE: Single-cell signature explorer
ssGSEA: Single sample GSEA
SAME-clustering: Single-cell Aggregated Clustering via Mixture Model Ensemble clustering
scCATCH: Single-cell cluster-based automatic annotation toolkit for cellular heterogeneity
SC3: Single-cell consensus clustering
scDaPars: Dynamic analysis of APA from single-cell RNA-seq
SCDE: Single-cell differential expression
scFBA: Single-cell flux balance analysis
scFEA: Single-cell flux estimation analysis
scLVM: Single-cell latent variable model
Scrublet: Single-cell remover of doublets
scRNA-seq: Single-cell RNA sequencing
scVDMC: Variance-driven multitask clustering of scRNA-seq data
SIMLR: SAingle-cell interpretation via multikernel learning
SLICER: Selective locally linear inference of cellular expression relationships
SNN: Shared nearest neighbor
SoptSC: Similarity matrix-based optimization for single-cell data analysis
Squidpy: Spatial quantification of molecular data in Python
SVM: Support vector machine
TSCAN: Tools for single cell analysis
t-SNE: t-distributed stochastic neighbor embedding
TF: Transcription factor
TPM: Transcript per million
TMM: Trimmed mean of M values
TRRUST: Transcriptional regulatory relationships unravelled by sentence-based text-mining
UMAP: Uniform manifold approximation and projection
UMI: Unique molecular identifier
UQ: Upper quartile
WGCNA: Weighted gene co-expression network analysis
ZIFA: Zero-inflated factor analysis
